# Treatment with a GnRH receptor agonist, but not the GnRH receptor antagonist degarelix, induces atherosclerotic plaque instability in ApoE^**−/−**^ mice

**DOI:** 10.1038/srep26220

**Published:** 2016-05-18

**Authors:** Anki Knutsson, Sabrina Hsiung, Selvi Celik, Sara Rattik, Ingrid Yao Mattisson, Maria Wigren, Howard I. Scher, Jan Nilsson, Anna Hultgårdh-Nilsson

**Affiliations:** 1 Department of Experimental Medical Science, Lund University, Lund, Sweden; 2 Department of Clinical Sciences Malmoe, Lund University, Malmoe, Sweden; 3 Sidney Kimmel Center for Prostate and Urologic Cancers, Memorial Sloan-Kettering Cancer Center and Weill Cornell Medical College, New York, NY

## Abstract

Androgen-deprivation therapy (ADT) for prostate cancer has been associated with increased risk for development of cardiovascular events and recent pooled analyses of randomized intervention trials suggest that this primarily is the case for patients with pre-existing cardiovascular disease treated with gonadotropin-releasing hormone receptor (GnRH-R) agonists. In the present study we investigated the effects of the GnRH-R agonist leuprolide and the GnRH-R antagonist degarelix on established atherosclerotic plaques in ApoE^**−/−**^ mice. A shear stress modifier was used to produce both advanced and more stable plaques in the carotid artery. After 4 weeks of ADT, increased areas of necrosis was observed in stable plaques from leuprolide-treated mice (median and IQR plaque necrotic area in control, degarelix and leuprolide-treated mice were 0.6% (IQR 0–3.1), 0.2% (IQR 0–4.4) and 11.0% (IQR 1.0-19.8), respectively). There was also evidence of increased inflammation as assessed by macrophage immunohistochemistry in the plaques from leuprolide-treated mice, but we found no evidence of such changes in plaques from control mice or mice treated with degarelix. Necrosis destabilizes plaques and increases the risk for rupture and development of acute cardiovascular events. Destabilization of pre-existing atherosclerotic plaques could explain the increased cardiovascular risk in prostate cancer patients treated with GnRH-R agonists.

Androgen deprivation therapy (ADT) is a well-established treatment for advanced prostate cancer. Treatment with gonadotropin-releasing hormone receptor (GnRH receptor) agonists is currently the most common approach for ADT. GnRH receptor agonists, such as leuprolide and goserelin, produce a decline in testosterone after an initial testosterone surge in the first 1–3 weeks of therapy^1^. However, ADT is also associated with metabolic side effects including increased body fat, dyslipidemia, hyperglycemia and insulin resistance^2^,^3^ which may increase the risk for diabetes and cardiovascular disease (CVD)^4^,^5^. Accordingly, recent meta-analysis based on studies involving a total of 129,802 ADT users and 165,605 controls demonstrate a 20% increase in cardiovascular events in patients treated with GnRH receptor agonists. GnRH receptor antagonists that block GnRH receptors in the anterior pituitary gland, represent an alternative approach for medical ADT. Treatment with GnRH receptor antagonists results in a more rapid decrease in testosterone production with castrate levels (≤0.5 ng/mL) reached within 1–3 days, no surge^6^, and similarly rapid elimination of the free forms of prostate-specific antigen (PSA) and kallikrein-related peptidase 2 (hK2) in blood^7^. Interestingly, recent observational studies suggest that GnRH receptor agonists and GnRH receptor antagonists may differ in respect to cardiovascular risk. Pooled data from randomized phase III/IIIb trials comparing the GnRH receptor antagonist degarelix with GnRH receptor agonists showed a 40% lower risk of a cardiac event or death with degarelix^8^. The difference in cardiovascular risk was primarily observed in patients with pre-existing CVD and was evident already after a few months of treatment. The mechanisms through which GnRH receptor agonists could contribute to increased cardiovascular risk on a cellular and molecular level remain unclear. However, the observations that the increase in cardiovascular risk occurs within months of the initiation of ADT^9^,^10^ and mainly affect patients with pre-existing CVD^11^,^12^ suggest a direct effect on established atherosclerotic lesions rather than an effect on metabolic factors. Most acute cardiovascular events, such as myocardial infarction and stroke, are caused by rupture of an atherosclerotic plaque leading to the formation of an occluding thrombus or emboli^13^. The factors that lead to plaque rupture have been extensively studied and include intra- and extracellular lipid accumulation, loss of smooth muscle cells, degradation of fibrous tissue, and development of a necrotic core^13^,^14^. Inflammation driven by macrophages and Th1-type T cells plays a major role in the destabilization process^15^. There has been little research on the extent as to which GnRH receptor agonists and antagonists can influence these processes. In the present study we investigated the effects of degarelix and leuprolide on atherosclerotic plaques in high-fat fed ApoE^**−/−**^ mice. We used a shear stress-modifying cast to generate both advanced and more stable plaque characteristics in the carotid artery. The results show that treatment with leuprolide, but not degarelix, cause development of necrotic cores in the more stable plaques. This could provide an explanation to the observed increased cardiovascular risk of GnRH receptor agonists.

## Results

**Figure 1:**
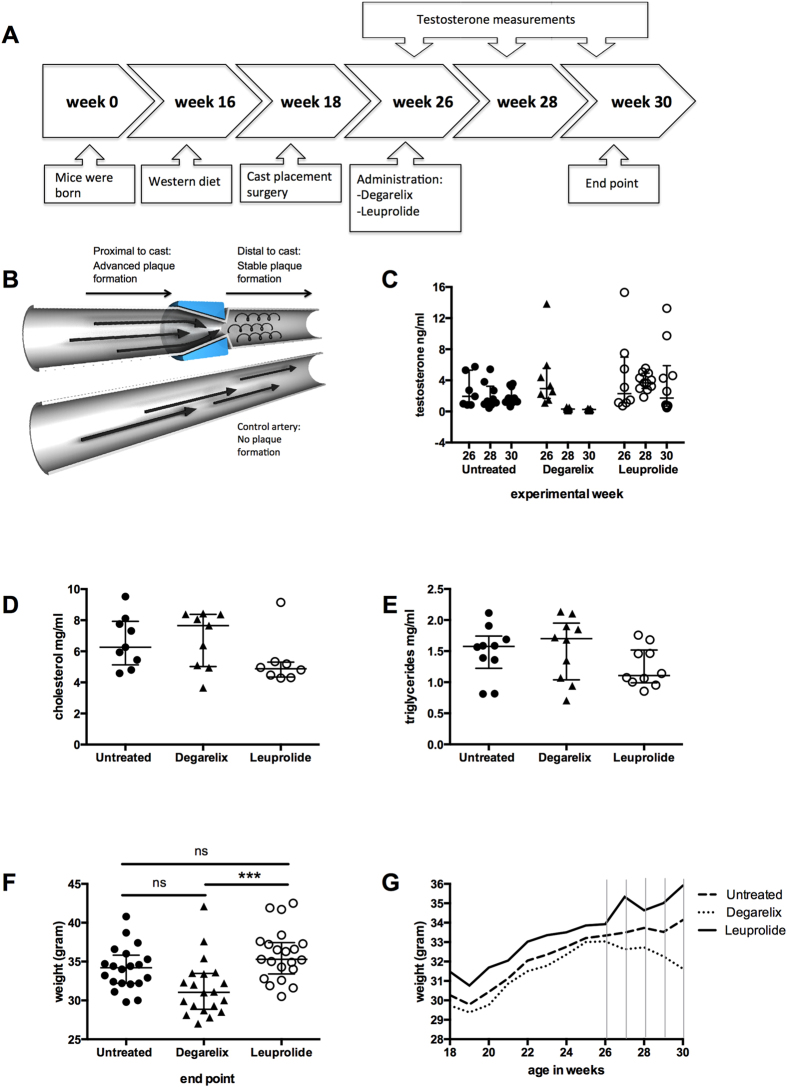
Schematic overview of the experimental model. ADT affects testosterone levels and body weight, but does not alter cholesterol or triglyceride levels. (**A**) Timeline over the experiment, (**B**) illustration of the shear stress-modifying cast showing where advanced and stable plaques are created, (**C**) testosterone measurements in plasma, (**D**) cholesterol levels, (**E**) triglyceride levels and (**F,G**) body weight in grams of control and leuprolide- and degarelix-treated mice. The left diagram demonstrates individual body weight of control and leuprolide- and degarelix-treated mice at the end point of the experiment. The right diagram shows a time course of mean values of the body weights. ***p < 0.001.

To investigate whether GnRH receptor agonists differ from the GnRH receptor antagonist in their effects on established atherosclerotic plaques, we used a shear stress-modifying cast to induce the formation of both advanced and stable plaques in the carotid artery of ApoE^**−/−**^ mice fed a high-fat diet. In this model, a cast placement around the artery results in the formation of advanced inflammatory plaques in the region proximal to the cast and more stable plaques distal to the cast (Fig. 1B)^16^. We began treatment with the GnRH receptor agonist leuprolide or the GnRH receptor antagonist degarelix 8 weeks after placement of the cast using untreated mice as controls (Fig. 1A). After 2 weeks of treatment, testosterone levels were significantly reduced in the degarelix group whereas no significant reduction could be observed in response to leuprolide (Fig. 1C). There was no significant difference in plasma cholesterol (Fig. 1D) or triglycerides between the groups (Fig. 1E). Treatment with degarelix was associated with a modest weight loss and by the end of the study these mice had significantly lower body weight in comparison to the control and leuprolide-treated mice (Fig. 1F,G).

### Expression of GnRH receptors in atherosclerotic plaques

**Figure 2:**
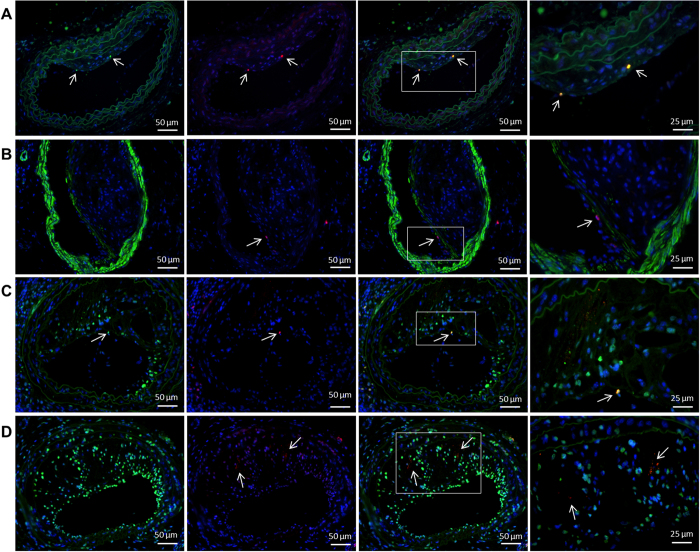
The GnRH receptor is expressed in the atherosclerotic plaque. Representative positive immune stainings of (**A**) the T cell marker CD3 (green) and the GnRH receptor (red) in carotid plaques. Co-localization of CD3 and GnRH receptor immunoreactivity is demonstrated by arrows. (**B**) Image of α-smooth muscle actin (green) and the GnRH receptor positive cells (red, demonstrated by arrows) in carotid plaques showing no co-localization. (**C**) Image of the macrophage marker F4/80 (green) and the GnRH receptor (red, demonstrated by arrows) in carotid plaques showing occasional co-localization. (**D**) Image of the macrophage marker F4/80 (green) and the GnRH receptor (red) showing a scattered staining without clear cell association, indicating positive GnRH receptor immunoreactivity on membrane remnants. Images represented as 20x magnification with 50 μm scale bars and 40x magnification with 25 μm scale bars.

We first used an immunofluorescence staining to investigate if GnRH receptors are expressed in atherosclerotic lesions. Cells expressing GnRH receptors were present in both stable and more advanced plaques. The GnRH receptor staining co-localized with CD3 positive cells demonstrating that the majority of the GnRH receptor expressing cells in the plaque were T cells (Fig. 2A). Indeed, all CD3 positive T cells detected in the plaques also demonstrated positive staining for GnRH receptors. The double staining for the GnRH receptor and the smooth muscle cell marker α-smooth muscle actin showed no co-localization (Fig. 2B). Although occasional cells demonstrated a double staining for the GnRH receptor and the macrophage marker F4/80 (Fig. 2C) most macrophages did not express the GnRH receptor (Fig. 2D). We also observed some co-localization between the GnRH receptor and the B cell marker CD45R/B220. However, since the CD45R/B220 positive cells also expressed CD3, they most likely represented activated T cells, which are known to express CD45R/B220. In the necrotic areas of the plaque some staining for the GnRH receptor could also be detected but here the staining pattern was scattered without clear cell association, indicating positive GnRH receptor immunoreactivity on membrane remnants (Fig. 2D, represented by arrows).

### Effect of degarelix and leuprolide on development of atherosclerosis and plasma cytokines

**Figure 3:**
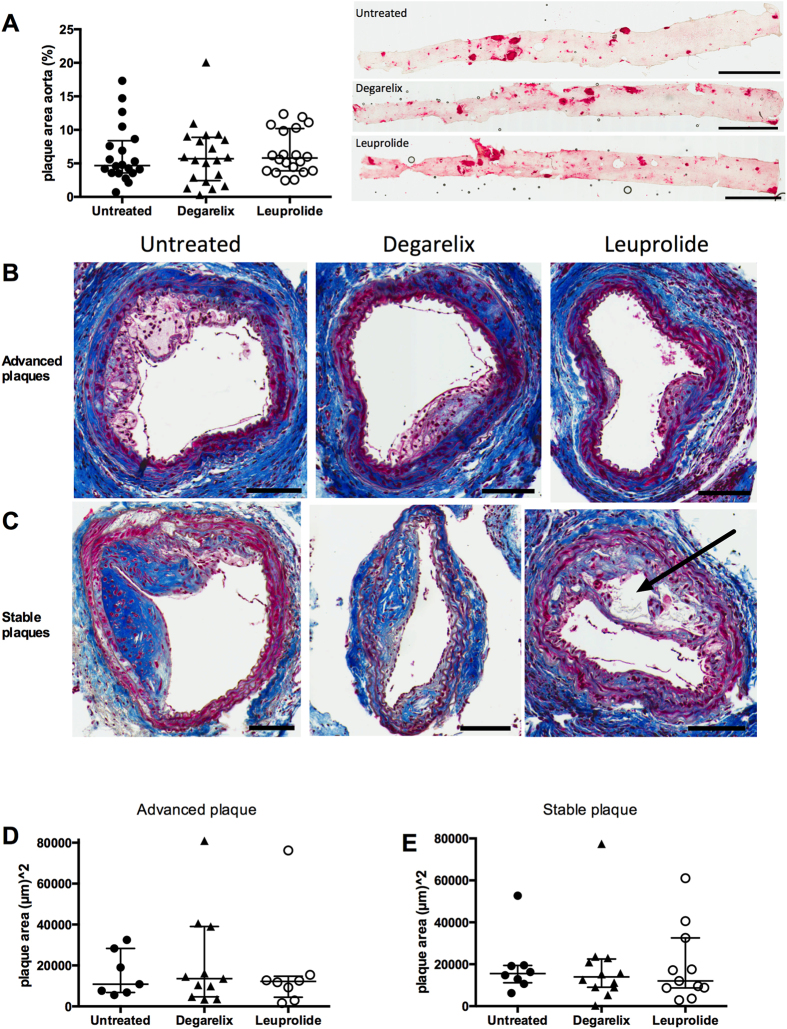
ADT did not alter the plaque burden but induced plaque necrosis in leuprolide treated mice. Quantification of (**A**) Oil Red O-stained flat preparations of aortas from control and degarelix- and leuprolide-treated mice. Scale bars represent 4 mm. Representative Masson’s trichrome stainings (fibrous tissue in blue and cells in red) of (**B**) advanced proximal plaques and (**C**) stable distal plaques from control, degarelix- and leuprolide-treated mice. Necrotic area is indicated by arrow. Scale bars represent 100 μm. Quantification of cross-sectional plaque area in (**D**) advanced plaques proximal to the cast and (**E**) stable plaques distal to the cast.

Assessment of overall atherosclerotic burden made by *en face* Oil Red O staining of the descending aorta showed no difference between the groups (Fig. 3A). There was also no difference in plaque area of advanced and stable lesions in the carotids (Fig. 3D,E). To determine whether treatment with leuprolide or degarelix was associated with activation of immune cells, we measured plasma levels of a number of T cell cytokines (IL-2, IL-4, IL-5, IL-6, IL-10, IL-12p70, IL-13, IL-17A) as well as the pro-inflammatory cytokine tumor necrosis factor-α. However, there was no difference between the groups (data not shown).

### Effect of degarelix and leuprolide on plaque vulnerability

**Figure 4:**
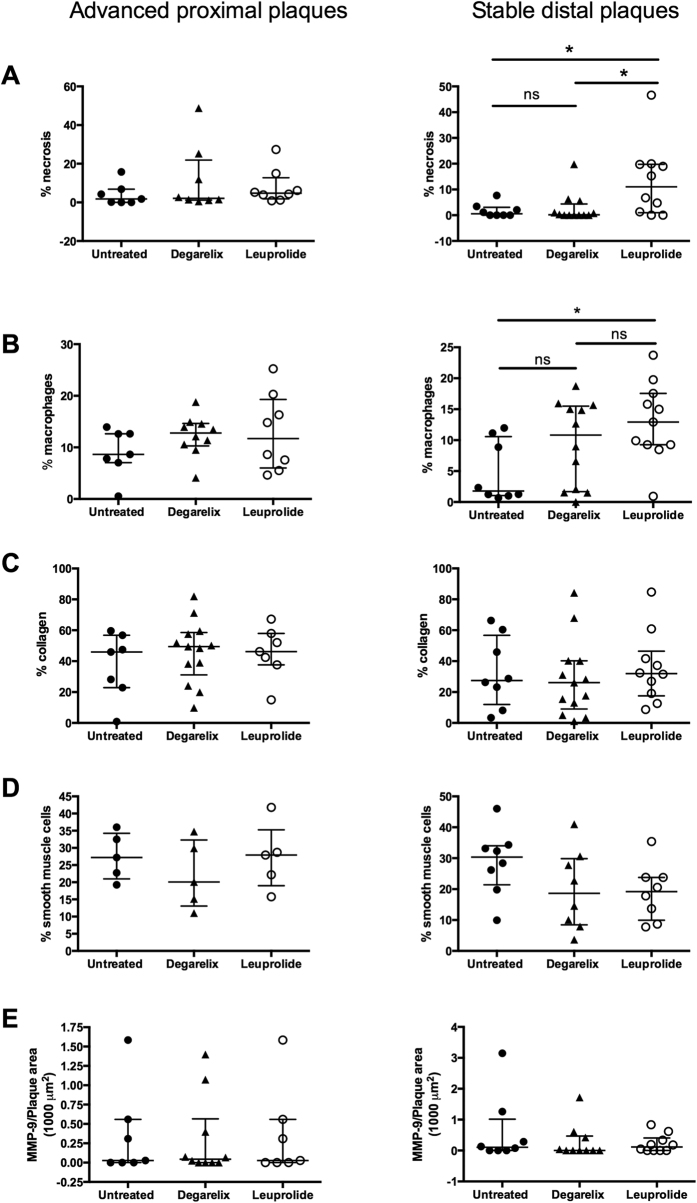
ADT did not alter the relative amount of collagen, smooth muscle cells or MMP-9 but did increase macrophage content and necrosis in stable distal plaques from leuprolide treated mice. Quantifications of staining for plaque (**A**) necrosis, (**B**) macrophages, (**C**) collagen, (**D**) smooth muscle cells and (**E**) MMP-9. *p < 0.05.

We next evaluated the effect of treatment with leuprolide or degarelix on plaque vulnerability by measuring necrotic core size, macrophage, smooth muscle cell and collagen content of stable and more advanced plaques. In stable plaques distal to the cast, the median necrotic area was 0.6% (IQR 0–3.1) in control mice, 0.2% (IQR 0–4.4) in degarelix-treated mice, but as high as 11.0% (IQR 1.0–19.8) in leuprolide-treated mice (Figs 3C and 4A). Hence, 5 out of 10 mice in the leuprolide group had necrosis that exceeded 10% of the plaque area, as compared with only 1 out of 12 mice in the degarelix group, and none out of 8 mice in the control group. However, 20% of the mice in the leuprolide group demonstrated no sign of distal plaque necrosis at all (corresponding values for the control and degarelix groups were 50% in both cases) suggesting heterogeneity in the response to the GnRH receptor agonist. In the proximal advanced plaques, which generally are associated with larger necrotic areas, the median necrotic area was 1.8% (IQR 0–6.8) in the control, 2.1% (IQR 1.2–21.8) in the degarelix-treated group and 4.8% (IQR 1.8–12.8) in the leuprolide-treated group with no statistical significance between the different groups (Figs 3B and 4A). Leuprolide-treatment was also associated with increased macrophage accumulation in stable plaques (Fig. 4B). There were no significant differences in plaque collagen (Fig. 4C) and smooth muscle cells (Fig. 4D) or MMP-9 (Fig. 4E) between the groups.

### Effect of degarelix and leuprolide on smooth muscle cell viability

**Figure 5:**
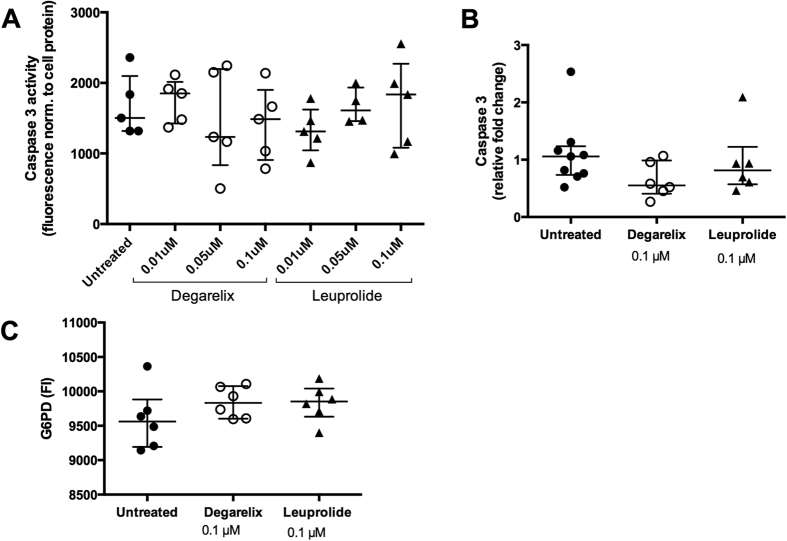
Degarelix and leuprolide do not directly affect smooth muscle cell apoptosis and cell viability. Cultured human coronary smooth muscle cells were treated for 24 hrs *in vitro* with the indicated concentrations of leuprolide or degarelix. (**A**) Caspase 3 activity was measured by fluorescence and the values normalized to cell protein where each dot represents one well. Data is representative of 3 independent experiments. (**B**) Expression of caspase 3 mRNA. Values are expressed as fold change of treated cells compared to untreated cells after normalization to housekeeping gene GAPDH. (**C**) Cell viability as assessed by release of glucose-6-phosphate dehydrogenase (G6PD).

To investigate the possibility that the increased presence of necrosis in distal plaques of leuprolide-treated mice was due to a direct effect of the drug on the viability of smooth muscle cells, we incubated cultured human coronary artery smooth muscle cells with leuprolide or degarelix for 24 hours. However, none of the treatments induced smooth muscle cell apoptosis as assessed by analyses of caspase 3 activity or mRNA expression (Fig. 5A,B) or cell necrosis as assessed by the release of glucose-6-phosphate dehydrogenase into the culture medium (Fig. 5C). Exposure of cultured human coronary artery smooth muscle cells to leuprolide or degarelix for 24 hours did not affect total cell protein levels suggesting a lack of effect on cell growth (data not shown).

## Discussion

A recent pooled analysis of six phase 3 randomized trials involving 2328 men with advanced prostate cancer show differences in cardiovascular risk among men treated with GnRH receptor agonists versus those treated with GnRH receptor antagonists, and provide additional evidence that treatment with GnRH receptor agonists is associated with increased incidence of cardiovascular events in patients with pre-existing CVD^8^. As most acute cardiovascular events are caused by rupture of atherosclerotic plaques^13^, this could suggest that treatment with GnRH receptor agonists contributes to plaque destabilization. In the present study, we used an established animal model in which atherosclerotic lesions are generated in carotid arteries by the combination of hypercholesterolemia and altered shear stress induced by a perivascular cast. The model induces formation of advanced plaques in the low shear stress region proximal to the cast and stable lesions in the oscillatory shear stress region distal to the cast^16^. Mice were treated with either the GnRH receptor agonist leuprolide or the GnRH receptor antagonist degarelix during four weeks to determine the short-term effects on plaque stability. Leuprolide-treatment was associated with evidence of significantly increased inflammation and the development of necrotic cores in the distal more stable plaques as opposed to that there was no evidence of these effects in response to treatment with degarelix, or in the proximal advanced plaques. Furthermore, there were no significant changes associated with either treatment in reference to plaque size, collagen content or the extent of atherosclerosis in the aorta. Our findings are in line with a recent study by Hopmans *et al.*^17^ demonstrating increased necrosis in aortic root atherosclerotic lesions in LDL receptor-deficient mice treated with leuprolide as compared with degarelix. However, there are some important differences in the design between the present study and that of Hopmans and coworkers. The latter study investigated the effect of long-term (4 months) treatment on early plaque development while we analyzed the effect of short-term (1 month) treatment on already established plaques. This difference may explain why the effect on plaque necrosis by leuprolide was more prominent in the present study as well as why we did not observe any effect on general atherosclerosis burden. Prostate cancer patients with pre-existing CVD are likely to have advanced atherosclerotic plaques. Against this background we evaluated the effect of leuprolide and degarelix in a mouse model in which late stage plaques of both advanced and stable phenotype can be generated by alteration of carotid shear stress. Notably, the necrosis that was observed in stable plaques from leuprolide treated animals in this model (around 10% of plaque area) was considerably larger than that observed by Hopmans and coworkers^17^ in aortic root plaques (around 1% of plaque area).

The mechanism through which treatment with a GnRH receptor agonist such as leuprolide may increase the risk for necrosis in atherosclerotic plaques remains to be fully characterized. The observation that no such effect was observed in response to treatment with the GnRH receptor antagonist degarelix suggests that activation of the GnRH receptor is important. Treatment with GnRH receptor agonists is known to down-regulate the expression of the receptor in the pituitary gland leading to a suppression of luteinizing hormone (LH), follicle-stimulating hormone (FSH) and testosterone levels. However, before this down-regulation occurs there is an initial increase in LH, FSH and testosterone levels due to an increased stimulation of the receptor. In contrast, GnRH receptor antagonists lead to a rapid inhibition of the receptor and down-regulation of LH, FSH and testosterone levels. The observation that treatment with leuprolide did not reduce testosterone levels in the present study suggests that GnRH receptors had not yet been down-regulated and remained responsive to stimulation with the agonist. We used an immunofluorescence staining to determine the presence of GnRH receptor expressing cells in atherosclerotic plaques and found that almost all cells expressing this receptor were T cells. Presence of GnRH receptors has previously been demonstrated on cultured T cells and shown to stimulate the activity of these cells^18^,^19^. Activation of GnRH receptors on T cells is also known to favor generation of the pro-inflammatory Th1-type T cells^20^ that aggravate atherosclerosis^21^. Collectively, these observations suggest a role for T cells in the destabilizing effect of GnRH agonists on atherosclerotic plaques. GnRH receptor expression could also be detected on some plaque macrophages. Accordingly, it cannot be excluded that activation of plaque macrophages could be involved. Macrophages have been attributed an important role in atherosclerotic plaque destabilization through the release of extracellular tissue degrading MMPs^22^. However, we did not observe an increased expression of MMP-9 or decreased collagen levels in the stable distal lesions. An effect dependent on activation of macrophages is also likely to have been more prominent in the advanced macrophage-rich proximal plaques rather than in the stable distal plaques. Moreover, Min *et al.* have reported evidence for a suppressive effect of the GnRH receptor on macrophage function^23^. Taken together these observations argue against a role for macrophage GnRH receptor activation in GnRH agonist-induced atherosclerotic plaque necrosis. Moreover, our immunofluorescence studies revealed that most GnRH receptor-expressing cells inside the plaques were not macrophages.

An interesting and unexpected finding of the present study was that increased plaque necrosis occurred only in the distal stable lesions. These lesions are generally more smooth muscle cell-rich than the more inflammatory proximal low shear stress plaques^16^. Although not statistically significant, we observed a trend towards decreased smooth muscle cell content in distal plaques of leuprolide-treated mice. GnRH receptor agonists, such as leuprolide, are a well-established treatment for leiomyoma, a benign uterine smooth muscle cell tumor. The leiomyoma-suppressing effect of GnRH receptor agonists has traditionally been attributed to inhibition of the pituitary-gonadal axis and ovarian steroid hormone production^24^,^25^, but there is also evidence of a direct effect of GnRH receptor activation in leiomyoma cells^26^,^27^. Leuprolide inhibits the proliferation of cultured myometrial smooth muscle cells^26^ and clinical studies have shown that treatment with leuprolide suppress proliferation and induce apoptosis in uterine leiomyoma^28^. The latter effect was evident after 4 weeks of treatment but vanished subsequently. It is an interesting possibility that the loss of smooth muscle cells and induction of plaque necrosis observed after 4 weeks of treatment with leuprolide in the present study also is the result of an activation of smooth muscle cell apoptosis. However, the present observation that incubation with leuprolide does not induce apoptosis or necrosis in cultured human coronary smooth muscle cells argues against a direct effect of the GnRH receptor agonist on smooth muscle cell viability. Furthermore, our immunofluorescence studies showed that the GnRH receptor was not expressed on smooth muscle cells in the plaque. The possibility that the GnRH receptor agonist induces apoptosis in plaque smooth muscle cells should be further elucidated in future experimental studies.

There are some limitations of the present study that should be considered. Most importantly results from animal models of atherosclerosis are not directly transferable to the clinical situation and the observation that treatment with GnRH receptor agonists may induce necrosis in atherosclerotic plaques requires confirmation in human studies. The mechanisms through which leuprolide-treatment caused plaque necrosis in ApoE^**−/−**^ mice also need to be further characterized as well as why only certain types of plaques are affected. The possible role of the decreased body weight in degarelix treated mice should be further investigated to determine if this is of importance for the different effect of leuprolide and degarelix on atherosclerotic plaque stability. Previous studies suggest that the decreased body weight of degarelix-treated mice is explained by hormonal effects leading to loss of adipose tissue^17^ but it remains to be clarified if this also can affect atherosclerotic plaque stability.

In conclusion, the present study demonstrates that treatment with the GnRH receptor agonist leuprolide is associated with induction of necrosis in certain types of atherosclerotic plaques, while no such effect could be observed in mice treated with the GnRH receptor antagonist degarelix. The biological mechanisms involved in the plaque destabilizing effect of the GnRH agonist remains to be fully characterized but are likely to involve T cells since they were the most common GnRH receptor expressing cells in the plaque. Plaque necrosis is considered to increase the risk for plaque rupture, the major cause for development of acute cardiovascular events, such as myocardial infarction and stroke. It is possible that this effect may explain the difference in cardiovascular risk between GnRH agonists and degarelix that has recently been observed in retrospective pooled analysis of randomized intervention trials^8^.

## Materials and Methods

### Animals and *in vivo* alteration of shear stress

Male ApoE^**−/−**^ mice (B6.129P2-*Apoe*^*tm1Unc*^/J, The Jackson Laboratory) were fed an atherogenic high cholesterol-diet (R638; maize starch, cocoa butter, casein, glucose, sucrose, cellulose flour, minerals, and vitamins; 17.2% protein, 21% fat [62.9% saturated, 33.9% unsaturated and 3.4% polyunsaturated], 0.15% cholesterol, 43% carbohydrates, 10% H_2_O, and 3.9% cellulose fibers) for the duration of 14 weeks starting at the age of 16-weeks. Two weeks post-onset of the diet, a perivascular shear stress-modifying cast was placed around the right common carotid artery to alter the pattern of hemodynamic flow, as described by Cheng *et al.*^16^. The cast creates two different patterns of hemodynamic flow. Proximal to the cast, lowered shear stress induces lesions with advanced plaque phenotype. Distal to the cast, oscillatory shear stress generates lesions with a more stable phenotype^16^. In short, the surgery was carried out under anesthesia with oxygen-carried isoflurane. Buprenorphine was administered subcutaneously at 0.1 mg/kg before and after surgery. During week 26, degarelix and leuprolide were given subcutaneously at 30 mg/kg and 0.47 mg/kg respectively; one group was left untreated as a control. At 30-weeks of age the mice were sacrificed by an overdose of isoflurane. All experiments involving mice were approved by the Malmö/Lund Ethical Committee for Animal Research (Sweden) and were carried out in accordance with the approved guidelines.

### Sample preparation and histological analyses

The mice were perfused with Histochoice, the right carotid arteries were taken out to determine the atherosclerotic burden and the left carotid arteries were used as controls. The carotid arteries were fixed in Histochoice, embedded in paraffin and sectioned (5 μm).

Carotid artery sections were stained with Accustain trichrome (Masson) (Sigma-Aldrich) according to the manufacturer’s instructions. Flat preparations of descending aortas were stained with 0.3% Oil Red O for 50 minutes and mounted with Mountquick (Daido Sangyo Co. LTD, Tokyo, Japan). Carotid artery sections were immunohistochemically stained using antibodies against Mac-2 (Cedarlane; Burlington, ON Canada), F4/80 (AbCam 60343), MMP-9 (AbCam 38898), Anti-smooth muscle myosin heavy chain 11 (AbCam 125884), Anti-Actin, α-Smooth Muscle (Sigma-Aldrich A2547), CD3 (AbCam 11089), CD45R/B220 (AbCam 64100) and the GnRH receptor (AbCam 183079). Sections were deparaffinized and rehydrated in xylene and a graded series of alcohols before heat-induced antigen epitope retrieval was performed (pH 6.0, 20 minutes). The ImmPRESS HRP anti-rat (mouse absorbed) and anti-rabbit Ig (Peroxidase) polymer detection kits (Vector Laboratories) were used for the staining procedures according to the manufacturer’s instructions. For immunofluorescence the same procedure was followed using a Cy3-conjugated secondary goat anti-rabbit rat IgG (Jackson) or a goat anti-rat IgG (Agrisera, DyLight®488 conjugated) or a biotinylated anti-mouse IgG (Vector Laboratories) in combination with fluorescein-streptavidin (Amersham) for detection instead of the HRP-rat secondary antibody from the ImmPRESS kit.

Lesion size is expressed as absolute size and necrotic areas are defined as areas not stained by Masson’s Trichrome and are calculated as a percentage of the plaque; presented as the mean value of four sections collected where each lesion was at its largest. Immunohistochemically stained sections (visualized with DAB) were scanned and digitalized using an Aperio ScanScope digital slide scanner (Scanscope Console v8.2.0.1263, Aperio Technologies, Inc., Vista, California, USA) and images of immunofluorescent stains were taken using a Zeiss Axiophot 2 with a Hamamatsu C4742-95 camera, an X-Cite series 120Q lamp (Lumen Dynamics) and Openlab 5 software (Improvision). Image analysis was performed using BioPix iQ software (BioPixAB, Gothenburg, Sweden).

### Plasma retrieval and analyses

Blood was collected by cardiac puncture and plasma retrieved by centrifugation at 3000 rpm for 15 min at 4 °C. The colorimetric assay Infinity Total Cholesterol (Thermo Scientific, Liverpool, UK) was used to quantify total plasma cholesterol and the Bio-Plex Pro Mouse Cytokine Assay (BIO-RAD) was used to quantify plasma cytokine concentrations of with IL-2, IL-4, IL-5, IL-6, IL-10, IL-12p70, IL-13, IL-17A and tumor necrosis factor-α (TNF-α). Both analyses were performed according to instructions from the manufacturer.

### *In vitro* analysis of apoptosis and necrosis

Human coronary SMCs (hCASMC) were maintained in medium 231 with smooth muscle growth supplement (both cells and supplements from ThermoFisher Scientific, Waltham, MA, US). For Caspase 3 activity measurements, 25 000 cells per well in a 48 well plate were grown for 1–2 days in supplemented medium. Cells were then serum starved for 24 hours before they were stimulated with the indicated concentrations of Degarelix or Leuprolide for 24 hours. Caspase 3 activity was measured in the protein lysate from each well using a fluorogenic caspase 3 assay kit according to the manufacturer’s instructions (BD Biosciences Franklin Lakes, NJ, US). Protein concentration was measured using Pierce BCA protein assay kit (ThermoFisher Scientific). For analysis of caspase 3 mRNA expression HCASMC were treated as above, washed with 4 °C PBS (Biochrom, Cambridge, UK) and homogenized in TRI-reagent (Sigma Aldrich, MI, USA) using an Omni tissue homogenizer. SYBR Green PCR kit (Qiagen, Hilden, Germany) and commercially available primers for CASP3 and the housekeeping gene GAPDH (Qiagen) was used for mRNA gene expression quantification. The reaction was carried out in a Viia7 Real-time PCR system (Bio-Rad, CA, USA) after activation for 5 min in 95 °C followed by 40 cycles in the conditions; denaturation for 10 s in 95 °C, annealing and extension for 30 s in 60 °C. The cycle number when fluorescence signal reached threshold (C_T_) was determined and results of gene expression were expressed as fold change of treated cells compared to untreated after normalization to housekeeping gene GAPDH. The cell supernatants were saved to investigate possible necrosis using the Vybrant cytotoxicity assay (Thermo Fisher, MA, USA), which was performed according to manufacturer’s advice.

### Statistics and software

Error bars represent median with interquartile range. Statistical analyses were performed using Kruskal-Wallis analysis of variance, followed by Dunn’s multiple comparison tests when appropriate. Statistics on necrotic measurements were performed with logged values. GraphPad Prism version 6.0 was used for the statistical tests (GraphPad Software, San Diego, California, USA, www.graphpad.com).

## Additional Information

**How to cite this article**: Knutsson, A. *et al.* Treatment with a GnRH receptor agonist, but not the GnRH receptor antagonist degarelix, induces atherosclerotic plaque instability in ApoE^−^/^−^ mice. *Sci. Rep.* **6**, 26220; doi: 10.1038/srep26220 (2016).

## References

[b1] Van Poppel H. LHRH agonists versus GnRH antagonists for the treatment of prostate cancer. Belgian J Med Oncol 4, 18–22 (2010).

[b2] Hakimian P. *et al.* Metabolic and cardiovascular effects of androgen deprivation therapy. BJU Int 102, 1509–1514 (2008). 18727614 10.1111/j.1464-410X.2008.07933.x

[b3] Saylor P. J. & Smith M. R. Metabolic complications of androgen deprivation therapy for prostate cancer. J Urol 181, 1998–2006 (2009). 19286225 10.1016/j.juro.2009.01.047 PMC2900631

[b4] Basaria S. Androgen deprivation therapy, insulin resistance, and cardiovascular mortality: an inconvenient truth. J Androl 29, 534–539 (2008). 18567642 10.2164/jandrol.108.005454

[b5] Nobes J. P., Langley S. E. & Laing R. W. Metabolic syndrome and prostate cancer: a review. Clin Oncol (R Coll Radiol) 21, 183–191 (2009). 19111451 10.1016/j.clon.2008.11.013

[b6] Klotz L. *et al.* The efficacy and safety of degarelix: a 12-month, comparative, randomized, open-label, parallel-group phase III study in patients with prostate cancer. BJU Int 102, 1531–1538 (2008). 19035858 10.1111/j.1464-410X.2008.08183.x

[b7] Ulmert D. *et al.* Rapid elimination kinetics of free PSA or human kallikrein-related peptidase 2 after initiation of gonadotropin-releasing hormone-antagonist treatment of prostate cancer: potential for rapid monitoring of treatment responses. Clin Chem Lab Med 50, 1993–1998 (2012). 22718641 10.1515/cclm-2011-0967 PMC3474140

[b8] Albertsen P. C. *et al.* Cardiovascular morbidity associated with gonadotropin releasing hormone agonists and an antagonist. Eur Urol 65, 565–573 (2014). 24210090 10.1016/j.eururo.2013.10.032

[b9] D’Amico A. V. *et al.* Influence of androgen suppression therapy for prostate cancer on the frequency and timing of fatal myocardial infarctions. J Clin Oncol 25, 2420–2425 (2007). 17557956 10.1200/JCO.2006.09.3369

[b10] Kintzel P. E., Chase S. L., Schultz L. M. & O’Rourke T. J. Increased risk of metabolic syndrome, diabetes mellitus, and cardiovascular disease in men receiving androgen deprivation therapy for prostate cancer. Pharmacotherapy 28, 1511–1522 (2008). 19025432 10.1592/phco.28.12.1511

[b11] Nanda A., Chen M. H., Braccioforte M. H., Moran B. J. & D’Amico A. V. Hormonal therapy use for prostate cancer and mortality in men with coronary artery disease-induced congestive heart failure or myocardial infarction. JAMA 302, 866–873 (2009). 19706860 10.1001/jama.2009.1137

[b12] Hayes J. H. *et al.* Androgen-suppression therapy for prostate cancer and the risk of death in men with a history of myocardial infarction or stroke. BJU Int 106, 979–985 (2010). 20230380 10.1111/j.1464-410X.2010.09273.x

[b13] Hansson G. K. Inflammation, atherosclerosis, and coronary artery disease. N Engl J Med 352, 1685–1695 (2005). 15843671 10.1056/NEJMra043430

[b14] Libby P., Ridker P. M. & Hansson G. K. Progress and challenges in translating the biology of atherosclerosis. Nature 473, 317–325 (2011). 21593864 10.1038/nature10146

[b15] Lichtman A. H., Binder C. J., Tsimikas S. & Witztum J. L. Adaptive immunity in atherogenesis: new insights and therapeutic approaches. J Clin Invest 123, 27–36 (2013). 23281407 10.1172/JCI63108 PMC3533280

[b16] Cheng C. *et al.* Atherosclerotic lesion size and vulnerability are determined by patterns of fluid shear stress. Circulation 113, 2744–2753 (2006). 16754802 10.1161/CIRCULATIONAHA.105.590018

[b17] Hopmans S. N., Duivenvoorden W. C., Werstuck G. H., Klotz L. & Pinthus J. H. GnRH antagonist associates with less adiposity and reduced characteristics of metabolic syndrome and atherosclerosis compared with orchiectomy and GnRH agonist in a preclinical mouse model. Urol Oncol 32, 1126–34 (2014). 25242517 10.1016/j.urolonc.2014.06.018

[b18] Chen H. F., Jeung E. B., Stephenson M. & Leung P. C. Human peripheral blood mononuclear cells express gonadotropin-releasing hormone (GnRH), GnRH receptor, and interleukin-2 receptor gamma-chain messenger ribonucleic acids that are regulated by GnRH *in vitro*. J Clin Endocrinol Metab 84, 743–750 (1999). 10022447 10.1210/jcem.84.2.5440

[b19] Tanriverdi F., Gonzalez-Martinez D., Hu Y., Kelestimur F. & Bouloux P. M. GnRH-I and GnRH-II have differential modulatory effects on human peripheral blood mononuclear cell proliferation and interleukin-2 receptor gamma-chain mRNA expression in healthy males. Clin Exp Immunol 142, 103–110 (2005). 16178862 10.1111/j.1365-2249.2005.02904.x PMC1809497

[b20] Cavanagh P. C. *et al.* Gonadotropin-releasing hormone-regulated chemokine expression in human placentation. Am J Physiol Cell Physiol 297, C17–27 (2009). 19369450 10.1152/ajpcell.00013.2009

[b21] Wigren M., Nilsson J. & Kolbus D. Lymphocytes in atherosclerosis. Clin Chim Acta 413, 1562–8 (2012). 22565046 10.1016/j.cca.2012.04.031

[b22] Libby P. *et al.* Macrophages and atherosclerotic plaque stability. Curr Opin Lipidol 7, 330–335 (1996). 8937525 10.1097/00041433-199610000-00012

[b23] Min J. Y., Park M. H., Lee J. K., Kim H. J. & Park Y. K. Gonadotropin-releasing hormone modulates immune system function via the nuclear factor-kappaB pathway in murine Raw264.7 macrophages. Neuroimmunomodulation 16, 177–184 (2009). 19246940 10.1159/000204231

[b24] Takeuchi H., Kobori H., Kikuchi I., Sato Y. & Mitsuhashi N. A prospective randomized study comparing endocrinological and clinical effects of two types of GnRH agonists in cases of uterine leiomyomas or endometriosis. J Obstet Gynaecol Res 26, 325–331 (2000). 11147718 10.1111/j.1447-0756.2000.tb01334.x

[b25] Kettel L. M. *et al.* Rapid regression of uterine leiomyomas in response to daily administration of gonadotropin-releasing hormone antagonist. Fertility and sterility 60, 642–646 (1993). 8405517 10.1016/s0015-0282(16)56214-1

[b26] Chegini N., Rong H., Dou Q., Kipersztok S. & Williams R. S. Gonadotropin-releasing hormone (GnRH) and GnRH receptor gene expression in human myometrium and leiomyomata and the direct action of GnRH analogs on myometrial smooth muscle cells and interaction with ovarian steroids *in vitro*. J Clin Endocrinol Metab 81, 3215–3221 (1996). 8784072 10.1210/jcem.81.9.8784072

[b27] Luo X., Ding L., Xu J., Williams R. S. & Chegini N. Leiomyoma and myometrial gene expression profiles and their responses to gonadotropin-releasing hormone analog therapy. Endocrinology 146, 1074–1096 (2005). 15604208 10.1210/en.2004-1384

[b28] Mizutani T., Sugihara A., Nakamuro K. & Terada N. Suppression of cell proliferation and induction of apoptosis in uterine leiomyoma by gonadotropin-releasing hormone agonist (leuprolide acetate). J Clin Endocrinol Metab 83, 1253–1255 (1998). 9543151 10.1210/jcem.83.4.4696

